# The Effects of Leaf Extracts of Four Tree Species on *Amygdalus pedunculata* Seedlings Growth

**DOI:** 10.3389/fpls.2020.587579

**Published:** 2021-01-13

**Authors:** Xiuqing Wang, Ruiqi Zhang, Jinxin Wang, Long Di, Huaibiao Wang, Ashim Sikdar

**Affiliations:** ^1^Institute of Soil and Water Conservation, Northwest A&F University, Yangling, China; ^2^College of Natural Resources and Environment, Northwest A&F University, Yangling, China; ^3^Key Laboratory of Plant Nutrition and the Agri-environment in Northwest China, Ministry of Agriculture, Yangling, China; ^4^Forestry Industry Development Office of Yulin City, Yulin, China; ^5^Forestry Station of Yuyang District of Yulin City, Yulin, China; ^6^Department of Agroforestry and Environmental Science, Sylhet Agricultural University, Sylhet, Bangladesh

**Keywords:** *Amygdalus pedunculata*, stereo-complex ecosystem, seed germination, seedling growth, tree species, vegetation reconstruction

## Abstract

Vegetation reconstruction is an urgent problem in fragile environment like coal mine subsidence areas. *Amygdalus pedunculata* is an important eco-economic shrub species that promotes wind prevention, sand fixation as well as soil and water conservation. The natural regeneration of pure *Amygdalus pedunculata* forests is difficult to achieve because of its low seed germination rate and weak seedling growth. A stereo-complex ecosystem could potentially promote the germination and seedling growth of *A. pedunculata* and establish a steady mixed plantation consisting of trees and shrubs. Here, laboratory and pot experiments were conducted to assess the effect of four tree species on morphological and physiological indexes of *A. pedunculata*. The laboratory experiment showed that *A. pedunculata* seed germination and seedling growth from Yuyang County (YC-1) and Shenmu County (SC-6) were higher when plants were treated with the aqueous leaf extracts of *Pinus sylvestris*, *Broussonetia papyrifera*, and *Pinus tabulaeformis* compared with *Populus simonii* at concentrations of 2.5% (E2.5) and 5% (E5). Furthermore, the donor leaf extract was more sensitive to YC-1 than to SC-6. The pot experiment showed that the E2.5 and E5 treatments with the aqueous leaf extracts on the three tree species had strong promoting effects of seedling length, root length, seedling fresh weight, root fresh weight, and ground diameter for YC-1. The activity of catalase of *A. pedunculata* seedlings first increased and then decreased, while the activity of peroxidase, superoxide dismutase, roots, and the contents of soluble protein and chlorophyll decreased; the opposite patterns were observed for malondialdehyde, soluble sugar, cell membrane permeability, and proline were the opposite. Synthetical allelopathic effect index values of the leaf extracts of the three species on YC-1 were as follows: *P. sylvestris* > *B. papyrifera* > *P. tabulaeformis* (E2.5 to E20). Therefore, *P. sylvestris* and *B. papyrifera* could be used to promote the growth of *A. pedunculata* seedlings as well as for the construction of mixed plantations in coal mine degradation areas. Generally, this study provides new insight into the creation of stereo-complex ecosystems (*P. sylvestris* + *A. pedunculata* and *B. papyrifera* + *A. pedunculata*) in arid fragile environment.

## Introduction

**FIGURE 1:**
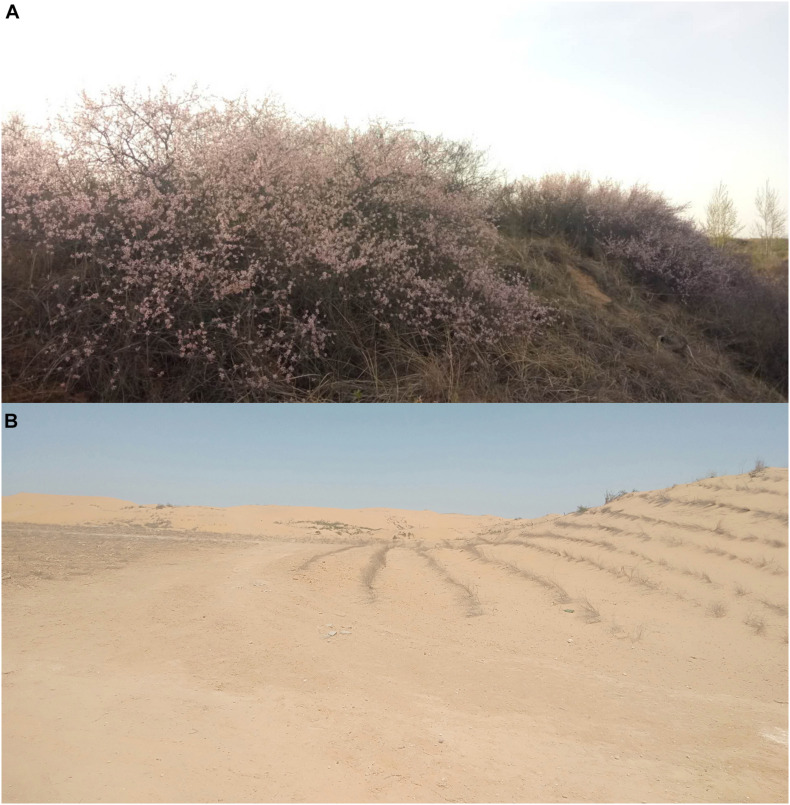
*Amygdalus pedunculata* grew in large quantities in Yulin before coal mining **(A)**. After coal mining and the partial replanting of *A. pedunculata*, the geomorphological characteristics changed extensively **(B)**.

*Amygdalus pedunculata* Pall. is an eco-economic shrub native to semi-arid and arid regions of northwestern China, especially Yulin (Browicz and Zohary, 1996; Yu et al., 2005). It is highly adaptable, light-loving, drought-tolerant, cold-tolerant, and disease-resistant which made it become a pioneer shrub species for afforestation in sand fixation. It can also be used as an ornamental shrub species and a source of honey in the early spring (Arman and Mohammad, 2011; Chu et al., 2013; Jing et al., 2013). It is also a woody oil-bearing shrub (Kester et al., 1991; Omirshat et al., 2009). Its biological and ecological characteristics, as well as its ecological and economic value, make it potentially useful for the development of bioenergy and the protection of the fragile ecological environment in the Yulin sand area (Eldridge et al., 2011; Caperta et al., 2014; Kleinhesselink et al., 2014). Successful planting of this species in the subsidence area of the Yulin coal mine could not only improve the ecological environment but could also potentially increase the income of local residents. However, previous studies have found that pure *A. pedunculata* forest can be difficult to grow, as the plants often grow exceptionally slowly or die during the seedling period. In the sand-fixing vegetation area, the health of *A. pedunculata* is affected by several factors: local natural conditions, the vulnerability of seedlings, moisture and nutrients in the sandy soil, low forest biodiversity, slow growth, low productivity, unstable ecosystems, difficult natural regeneration, and desertification in mining areas (Li et al., 2007; Kurosaki et al., 2011). These factors have hindered the growth of *A. pedunculata* plantations to some extent as the ecological environment continues to worsen. It is therefore imperative to find ways of cultivating *A. pedunculata* seedlings’ healthy development improve their ecological function and restore ecological stability. *A. pedunculata* developed largely in the Yulin region before mining. *A Pedunculata* original development is seen in Figure 1A and geomorphological characteristics after coal mining and existing partially replanted status of *A. pedunculata* in Figure 1B.

The survival rate of the seedling stage is key to the success of afforestation (Zhang et al., 2013). A single constructed artificial ecosystem often has incomplete ecological functions and is not sustainable long-term (Gamfeldt et al., 2013; Carnol et al., 2014; Mao et al., 2016). Stereo-complex ecosystems consisting of two components, such as mixtures of trees and shrubs, are beneficial for the growth and development of each element in the system (Nadrowski et al., 2010; Ruslandi et al., 2017). Such compound systems can improve the ecological conditions of stands, increase the amount of litter, and improve soil fertility (Scherer-Lorenzen et al., 2005; Santana et al., 2016). As the natural regeneration of *Amygdalus pedunculata* pure forests is difficult to accomplish because of its low seed germination rate and weak seedling growth, a stereo-complex ecosystem could be used to promote the germination and seedling growth of *A. pedunculata* and establish a steady mixed plantation consisting of trees and shrubs. Establishing more diverse plantations has been shown to improve the economic (Gamfeldt et al., 2013), ecological (Paquette and Messier, 2011), and cultural ecosystem services provided by plantation (Verheyen et al., 2016), especially sustainable and stable mixed plantations. However, few studies have been conducted on stereo-complex ecosystems consisting of woody plant species, especially with trees and shrubs in sandy areas.

The success of vegetation reconstruction depends to a large extent on the selection of plant species (Shi et al., 2011). To form multi-species and multi-level composite systems, a combination of trees and shrubs with fast growth, strong adaptability, and high resistance to stress should be selected to maximize the ecological and economic benefits (Keren et al., 2017). The dominant species in the tree layer influence the structure and function of the forest ecosystem. Windbreak, sand-fixing forests, and soil and water conservation forests can effectively improve soil structure, regulate local microclimates, protect undergrowth shrubs, and facilitate the growth of grass (Berendse, 1981; Crick and Grime, 1987). *Pinus sylvestris* var. and *Populus simonii* Carr. are a common native tree species in Yulin, Shaanxi Province. *P. sylvestris* is an evergreen coniferous tree species while *P. simonii* is a deciduous broad-leaved tree species in sandy areas of northern China (Zhao et al., 2010; Song et al., 2016). *Broussonetia papyrifera* Linn. is a new, fast-growing deciduous broad-leaved tree species (Zheng et al., 2008) while *Pinus tabulaeformis* Carr. is one of the most important evergreen coniferous tree species on the Loess Plateau (Liu et al., 2016). Four tree species are known to be highly resistant to drought, cold, and heat stress and show wide tolerance of windy, sandy, and barren conditions; these four species are also tall, long-lived species that could provide economically important resources in Yulin, an arid region of China (Chen and Cao, 2014; Meng et al., 2016; Cao et al., 2017; Yao et al., 2017), as animal husbandry and forestry are the major sources of income of the local people. Several problems, namely nutrient-deficient sandy soils and plant communities composed of single species, currently impede the construction of artificial pure forest of *A. pedunculata*. The construction of mixed plantations could help solve these problems.

Our previous study has shown that some shrubs and herbs can induce seed germination and seedling growth in *A. pedunculata* (Wang et al., 2018; Zhang et al., 2018). Trees are an important part of mixed plantations; however, few studies have assessed the ability of trees to facilitate *A. pedunculata* seed germination and seedling growth. In this study, we selected four additional suitable trees from the Mu Us Desert to study their effects on the morphological, physiological, and biochemical indicators of *A. pedunculata*. We first considered the hypothesis that a stereo-complex ecosystem consisting of trees and shrubs is conducive to the seedling settlement of *A. pedunculata*. The objectives of this study were to (1) leaf extracts of the four tree species affect the seed germination and seedling growth of *A. pedunculata*, (2) determine which tree species were optimal for promoting the growth of *A. pedunculata*, and (3) characterize variation in the physiological and biochemical indicators of *A. pedunculata* growth among the four tree species. This study provides new insight into the effect of the leaf extract of four tree species on *Amygdalus pedunculata* seedling growth as well as the potential utility of combining tree species for *A. pedunculata* artificial mixed forest construction. This study indicates that *A. pedunculata* plantations in arid and semi-arid regions could possibly be used to improve the ecological environment and promote local social and economic development.

## Materials and Methods

### Sample Collection and Extract Preparation

The fresh leaves of four tree species (*P. sylvestris*, *B. papyrifera*, *P. simonii*, and *P. tabulaeformis*) and two types *A. pedunculata* seeds (YC-1 was collected from Yuyang County and SC-6 from Shenmu County (SC) which were the representative *A. pedunculata* seeds in Yulin City, Shaanxi Province, China. The seeds were provided by the Yuyang District Forestry Station. *P. sylvestris*, *B. papyrifera*, *P. simonii*, and *P. tabulaeformis* leaves were collected after the growing season in October and November in 2017 at Bulanghe of Yulin.

Aqueous leaf extracts were prepared following the method of Le Rouzic et al. (2016). Briefly, 25, 50, 100, 150, and 200 g of dry leaf powder were soaked in 1000 mL of distilled water at room temperature for 48 h. After centrifugation at 3500 rpm for 20 min with a centrifuge (TG18G-II, Hunan Kaida, China), five concentrations of aqueous leaf extracts were obtained [2.5 (E2.5), 5 (E5), 10 (E10), 15 (E15), and 20% (C20)], and were stored at 4°C until use.

### Laboratory Experiments

The experimental methods for seed germination and seedling growth followed Mahboobi and Heidarian (2016), and specific operating parameters were obtained as previously described in Wang et al. (2018).

#### Seed germination experiment

*Amygdalus pedunculata* seeds (kernels, YC-1 and SC-6) were surface-sterilized by immersion for 15 min in 1% KMnO_4_ (potassium permanganate). The seeds were then rinsed with sterile distilled water and air-dried in a clean bench. A Petri dish (*d* = 12 cm) was lined with two layers of filter paper, and each Petri dish had 25 seeds. During the experiment 5 mL of sterile distilled water [0 (CK)] or five different concentrations [2.5 (E2.5), 5 (E5), 10 (E10), 15 (E15), and 20% (E20)] of aqueous leaf extracts of four tree species were added daily for both YC-1 and SC-6. Each treatment had four replications, and 168 Petri dishes overall (80 dishes with leave extract and 4 with pure water for both YC-1 and SC-6) for seed germination experiment. The prepared Petri dishes were arranged in a completely randomized design and were kept in an illuminated incubator (ZGZ-550D, Shanghai Binglin Electronic Technology Co., Ltd., Shanghai, China) for 10 days. The germination potential (GP) is the percentage of seed germination number on the third day to the total seeds number. Germination rate (GR) is the percentage of seed germination number on the 10th day to the total seed number. The germination index (GI) was calculated by the following equation.

$$\mathrm{Germination}\,\mathrm{index}\,\left(G\,I\right)=\sum \left(\frac{G\,t}{D\,t}\right)$$

where *Gt* is the number of seeds emerging on a given day, and *Dt* is the time after setting the seeds for germination.

#### Seedling growth experiment

After the germination test, *A. pedunculata* seedlings (60 strains per treatment) were cultivated in plastic plug holes (32 holes). Each hole was filled with 10 g of sterilized vermiculite and perlite (1:1), 10 mL of sterile distilled water [0 (CK)], or different concentrations of aqueous leaf extracts (E2.5, E5, E10, E15, E20) every 5 days. All treatments had four replications. All sample were randomly selected in every treatment to measure its morphological growth until it cultured in an artificial climate chamber for 30 days. Seedling length (SL) and root length (RL) were measured with ruler; seedling fresh weight (SFW) and root fresh weight (RFW) were measured with a precision balance.

### Pot Experiment

A pot experiment for seedling growth of *A. pedunculata* (YC-1) with the aqueous leaf extract of three tree species (*P. sylvestris*, *B. papyrifera*, and *P. tabulaeformis*) was conducted to further verify the results of the laboratory experiment. Surface soil was collected (20 cm) in the coal mine subsidence test fields in Yulin City. The chemical properties of the soil were as follows: pH 7.56, organic matter content 2.42 g kg^–1^, available nitrogen 85.23 mg kg^–1^, available phosphorus 11.34 mg kg^–1^, and available potassium 113.52 mg kg^–1^. YC-1 seeds were sown in the pots (14.5 cm in diameter and 23.5 cm deep) with 10 seeds per pot, and each pot contained 5 kg of soil. They were thinned to three plants per pot after growing for 30 days. During seedling growth, each group was treated with 50 mL of water [0 (CK)] or different concentrations of aqueous leaf extracts (E2.5, E5, E10, E15, E20) everyday respectively. Each treatment had four replications. The pot experiment was conducted for 8 months (2018.03–2018.10), and there were 64 pots in total. For measurements of plant biomass, plant samples were randomly selected in each treatment and were carefully washed with water to remove debris; different plant parts (underground and aboveground) were separated accordingly. Plant samples were initially dried in an oven for 20 min at 105°C, followed by further drying at 80°C; and finally, dry biomass of different plant parts was determined. Seedling length (SL) and root length (RL) were measured by a ruler. Seedling dry weight (SDW) and root dry weight (RDW) were measured with a precision balance; ground diameter (GD) was measured with digital vernier calipers.

The physiological and biochemical characteristics of *A. pedunculata* seedlings were also determined. Enzyme activity [specifically, the activities of catalase (CAT), peroxidase (POD), and superoxide dismutase (SOD)], malondialdehyde (MDA), root activity (RA), and chlorophyll (CHL) in plants were assessed (Wang et al., 2018). Soluble sugar content (SS) was determined by the anthrone colorimetry method (Bradford, 1976). Proline content (PRO) was determined using the acidic indene three ketone method (Bradford, 1976). Soluble protein content (SP) was determined using the Coomassie brilliant blue G-250 dyeing method (Yang et al., 2012). Cell membrane permeability (CMP) was determined by a conductivity meter (DDSJ-308A, Shanghai, China). *CMP* was first determined by the conductivity meter after fresh leaf tissue (0.2 g) and distilled water (10 mL) had been mixed for 12 h. The test tube was then placed into a boiling water bath for 30 min. After the test tube cooled, the determination was performed again by the same conductivity meter.

### Synthetical Allelopathic Effect Index (SE)

To determine the effects of different plant species on *A. pedunculata*, statistical comparisons among treatments were described by the indices of allelopathic effects (RI) which measures each treatment response (T) relative to its control (C). RI was defined as 1-(C/T) (T ≥ C) or T/C-1 (T < C) (Williamson and Richardson, 1988). Synthetical allelopathic effect index (SE) was used to describe the synthesize impact of each species on *A. pedunculata* which was determined as the arithmetic average of RI values of the test items measured by the donor (leaf extracts) on the same receptor *(A. pedunculata)*.

### Statistical Analysis

All data were analyzed using SPSS 22.0 (SPSS Inc., Chicago, IL, United States) and expressed as mean ± SD. ANOVAs, along with Duncan’s multiple range test (*P* < 0.05), were used to assess differences between treatments. Correlation analyses were used to study the relationships between different indicators. Figures were created with OriginPro 9.0 (Origin Lab Corporation, MA, United States) and HemI 1.0 (The CUCKOO Workgroup, Wuhan, China).

## Results

### Effects of Tree Aqueous Leaf Extracts on Seed Germination of *A. pedunculata*

In E2.5 and E2, the GP, GR, and GI of YC-1 all significantly increased (*P* < 0.05) under *P. sylvestris* and *P. tabulaeformis* leaf extracts (Supplementary Table 1). For both *P. sylvestris* and *P. tabulaeformis*, GP, GR, and GI peaked in E2.5 and decreased in other treatments; the decrease in these indicators was greater at higher concentrations. The three indicators were lower in E15 and E20 than in CK. The GR and GI peaked under the *P. sylvestris* E2.5 treatment, and the GR and GI were increased by 14.81% and 28.21% compared with the control. GP attained its maximum value under the *P. tabulaeformis* E2.5 treatment, which was increased by over 30% (Supplementary Table 1). However, the GP, GR, and GI of SC-6 showed a different trend, which peaked in the *P. tabulaeformis* E10 treatment and showed significant increases of 21.21%, 12.05%, and 28.03% (*P* < 0.05), respectively, compared with the control (Supplementary Table 2). The three indicators were all less than the control under the influence of the leaf extracts of the other two species (*B. papyrifera*, *P. simonii*), except the GP of E2.5 *B. papyrifera*.

### Effects of Tree Aqueous Leaf Extracts on Seedling Growth of *A. pedunculata*

**FIGURE 2:**
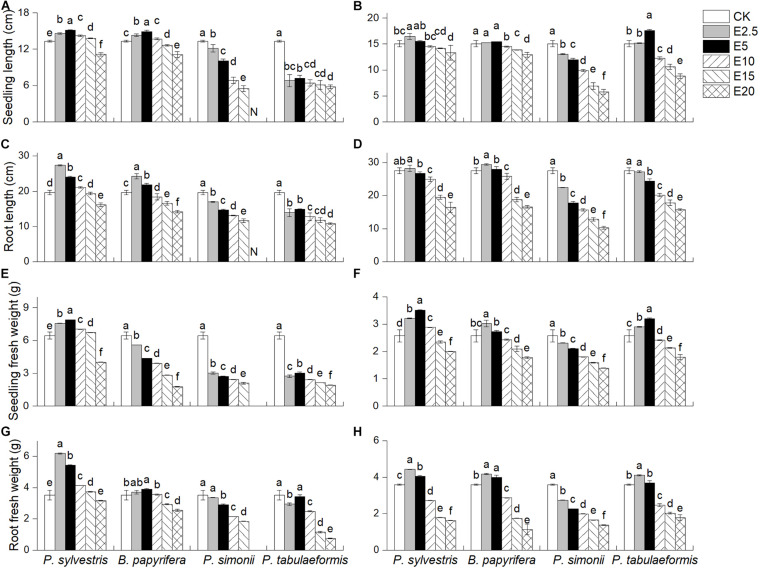
Effect of six different concentrations of aqueous leaf extracts [0% (CK), 2.5% (E2.5), 5% (E5), 10% (E10), 15% (E15), and 20% (E20)] collected from the donor species—*P. sylvestris*, *B. papyrifera*, *P. simonii*, and *P. tabulaeformis*—on **(A,B)** seedling length, **(C,D)** root length, **(E,F)** seedling fresh weight, and **(G,H)** root fresh weight of *A. pedunculata* (YC-1 and SC-6). “N” indicates that all plants had died; consequently, no relevant indicators were detected. Different letters in the same column indicate significant differences among different concentrations of aqueous leaf extracts from the same tree at the 0.05 level. Vertical bars represent standard deviation (*n* = 4).

*Pinus sylvestris* led to significantly increases in SL, RL, SFW, and RFW of YC-1 (Figures 2A,C,E,G) and SC-6 under E2.5 (Figures 2B,D,F,H). However, *P. simonii* and *P. tabulaeformis* reduced the four indicators, and the indicators decreased as the extract concentration increased. *P. tabulaeformis* significantly increase the SL, RFW, and SFW of SC-6 seedlings at some concentrations. RFW peaked in E5, whereas SL and SFW reached at E10. *B. papyrifera* in E2.5 and E5 significantly improved the RL and RFW of seedlings both for YC-1 and SC-6. The seedling growth indicators were all significantly less than the control when the leaf extract concentration was at its highest (E20) for every species.

### Effect on Morphological Growth Responses of *A. pedunculata*

To further verify the results of the indoor experiment, we carried out a pot experiment. The four trees were shown to have allelopathic and concentration effects on the growth of *A. pedunculata* seedlings. The E2.5 and E5 treatments with the aqueous leaf extracts of the three tree species had strong promoting effects (*P* < 0.05) of SL, RL, SDW, RDW, and GD for YC-1 (*P. sylvestris* extracts, E5, 23.32%, 21.91%, 35.75%, 25.16%, and 29.39% increases, respectively; *B. papyrifera* extracts, E5, 17.44%, 16.98%, 27.70%, 17.02%, and 19.71% increases, respectively; and *P. tabulaeformis* extracts, E2.5, 1.73%, 5.24%, 22.90%, 5.24%, and 13.98% increases, respectively) (Table 1). In addition, the promoting effects of the E15 and E20 treatments with the aqueous leaf extracts of the three tree species on seedling growth of YC-1 were reduced.

**TABLE 1:** Effect of different concentrations of the aqueous leaf extracts of three tree species on the seedling growth of *Amygdalus pedunculata* of YC-1.

Tree species	Concentrations (%)	Indicators (YC-1)				
		Seedling length	Root length	Seedling dry weight	Root dry weight	Stem diameter
		(cm)	(cm)	(g)	(g)	(mm)
*Pinus*	CK	12.72 ± 0.54^c^	30.14 ± 0.26^d^	2.82 ± 0.05^cd^	7.46 ± 0.06^d^	1.86 ± 0.02^e^
*sylvestris*	E2.5	14.19 ± 0.75^b^	35.19 ± 0.31^b^	3.17 ± 0.05^b^	8.71 ± 0.08^b^	2.15 ± 0.01^b^
	E5	15.69 ± 0.53^a^	37.64 ± 0.37^a^	3.82 ± 0.15^a^	9.33 ± 0.11^a^	2.41 ± 0.01^a^
	E10	13.54 ± 0.47^bc^	33.81 ± 0.27^c^	2.99 ± 0.05^bc^	8.37 ± 0.07^c^	2.07 ± 0.01^c^
	E15	10.73 ± 0.51^d^	30.53 ± 0.76^d^	2.77 ± 0.12^d^	7.55 ± 0.19^d^	1.9 ± 0.02^d^
	E20	7.28 ± 0.48^e^	27.01 ± 1.19^e^	1.91 ± 0.24^e^	6.68 ± 0.3^e^	1.69 ± 0.04
*Broussonetia*	CK	12.72 ± 0.54^b^	30.14 ± 0.26^b^	2.82 ± 0.05^b^	7.46 ± 0.06^b^	1.86 ± 0.02^d^
*papyrifera*	E2.5	13.78 ± 1.30^ab^	34.05 ± 0.51^a^	3.24 ± 0.39^a^	8.42 ± 0.13^a^	2.12 ± 0.01^b^
	E5	14.94 ± 0.90^a^	35.25 ± 0.25^a^	3.59 ± 0.17^a^	8.72 ± 0.07^a^	2.23 ± 0.01^a^
	E10	12.94 ± 0.66^b^	31.24 ± 0.47^b^	3.35 ± 0.22^a^	7.73 ± 0.12^b^	1.93 ± 0.01^c^
	E15	10.23 ± 0.31^c^	27.6 ± 1.87^c^	2.44 ± 0.32^c^	6.83 ± 0.46^c^	1.65 ± 0.03^e^
	E20	7.68 ± 1.21^d^	25.16 ± 0.81^d^	2.22 ± 0.06^c^	6.22 ± 0.20^d^	1.57 ± 0.02^f^
*Pinus*	CK	12.72 ± 0.54^a^	30.14 ± 0.26^c^	2.82 ± 0.05^b^	7.46 ± 0.06^c^	1.86 ± 0.02^b^
*tabulaeformis*	E2.5	12.94 ± 0.19^ab^	31.71 ± 0.36^b^	3.46 ± 0.06^a^	7.85 ± 0.09^b^	2.12 ± 0.1^a^
	E5	12.49 ± 0.15^a^	32.58 ± 0.22^a^	2.65 ± 0.11^b^	8.06 ± 0.05^a^	1.91 ± 0.01^b^
	E10	11.11 ± 0.12^b^	29.06 ± 0.28^d^	2.26 ± 0.17^c^	7.19 ± 0.07^d^	1.83 ± 0.02^bc^
	E15	9.74 ± 0.17^c^	26.07 ± 0.45^e^	2.2 ± 0.05^c^	6.45 ± 0.11^e^	1.71 ± 0.03^c^
	E20	8.32 ± 0.35^d^	23.24 ± 0.49^f^	1.86 ± 0.14^d^	5.75 ± 0.12^f^	1.58 ± 0.02^d^

*Mean ± standard deviation, *n* = 4.CK, 0%; E2.5, 2.5%; E5, 5%; E10, 10%; E15, 15%; E20, 20%. Different lowercase letters among treatments indicate significant differences at the 0.05 level.*

### Physiological and Biochemical Characteristics of *A. pedunculata* Seedlings

**FIGURE 3:**
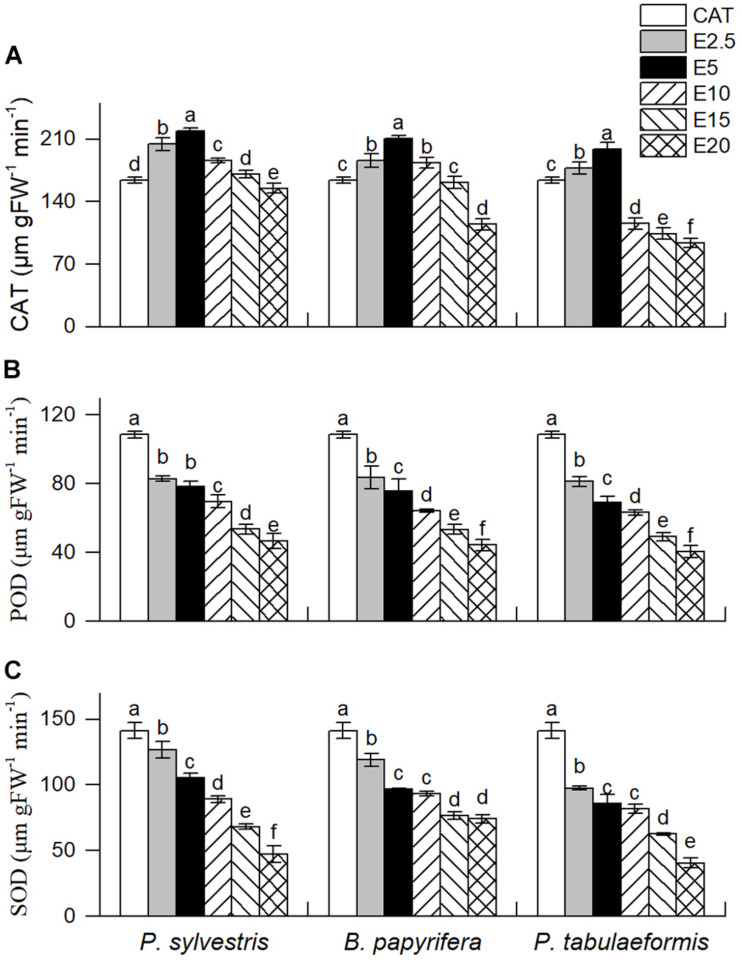
Effect of six different concentrations of aqueous leaf extracts [0% (CK), 2.5% (E2.5), 5% (E5), 10% (E10), 15% (E15), and 20% (E20)] collected from the donor species—*P. sylvestris*, *B. papyrifera*, and *P. tabulaeformis*—on the activities of **(A)** catalase (CAT), **(B)** peroxidase (POD), and **(C)** superoxide dismutase (SOD) of YC-1. Different letters in the same column indicate significant differences among different concentrations of aqueous leaf extracts from the same tree at the 0.05 level. Vertical bars represent standard deviation (*n* = 4).

Dominant species control their survival and expansion in plant communities by influencing the physiological and ecological characteristics of companion species (Ding et al., 2007). The CAT activity of YC-1 seedlings showed an initial increase followed by a decrease as the concentration of the aqueous leaf extracts increased (Figure 3A). However, the aqueous leaf extracts of the three tree species led to decreases in the activity of POD and SOD of YC-1 (*P. sylvestris* extracts, E2.5, 23.62% and 10.12% decreases; *B. papyrifera* extracts, E2.5, 23.01% and 15.72% decreases; and *P. tabulaeformis* extracts, E2.5, 25.15% and 30.87% decreases, *P* < 0.05) at all concentrations (Figures 3B,C).

Treatments with the three tree species had a superior ability to increase (*P* < 0.05) the contents of MDA, SS, CMP, and PRO of YC-1 (*P. sylvestris* extracts, E20, 73.14%, 88.21%, 63.94%, and 144.40% increases, respectively; *B. papyrifera* extracts, E20, 62.41%, 85.17%, 64.94%, and 197.25% decreases, respectively; and *P. tabulaeformis* extracts, E20, 369.15%, 82.24%, 67.85%, and 210.03% decreases, respectively) at all concentrations (Supplementary Table 3). Treatment with *P. sylvestris*, *B. papyrifera*, and *P. tabulaeformis* extracts on the SP contents of YC-1 decreased by 4.52%, 7.51%, and 15.65% under E2.5, respectively, and CHL content decreased by 47.31%, 52.14%, and 53.80% under E2.5, respectively (Supplementary Table 3). However, RA showed an initial increase (E2.5 and E5) followed by a decrease (E10 to E20) as the concentrations of aqueous leaf extracts increased (Supplementary Table 3). Treatment with *P. sylvestris*, *B. papyrifera*, and *P. tabulaeformis* extracts resulted in RA increases of 21.50%, 16.69%, and 15.02% of YC-1 under E5, respectively.

Thus, the aqueous leaf extracts of *P. sylvestris* and *B. papyrifera* did not exceed their own range for the degree of membrane damage of YC-1, demonstrating that there was a stronger promoting effect on YC-1 seedlings in terms of light absorption and utilization efficiency; the root system of YC-1 was also more developed and vigorous, which improved seedlings growth.

### The Correlation Between Process Variables and Morphological Parameters

**FIGURE 4:**
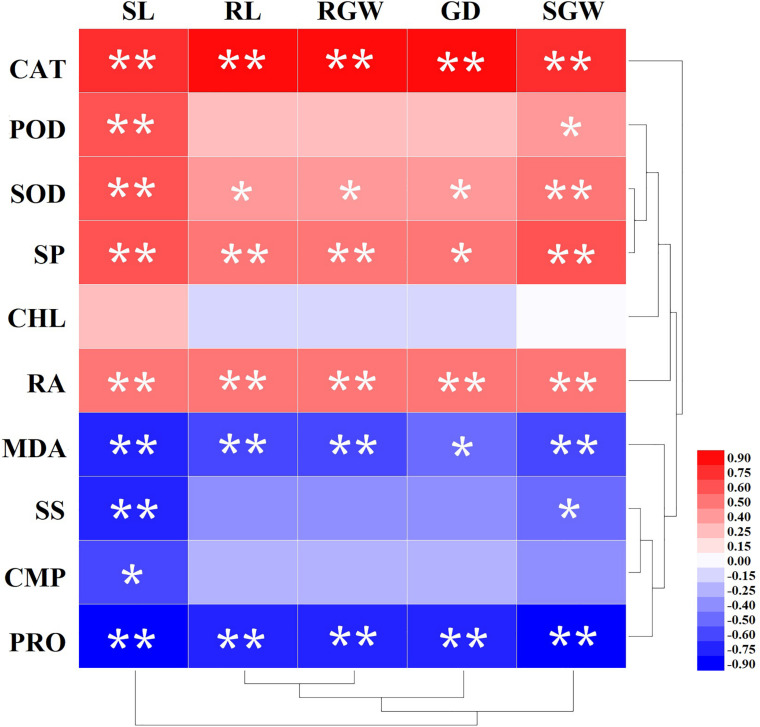
Correlation analysis of *Amygdalus pedunculata* growth and physiological and biochemical indexes. Two stars indicate that indexes showed a highly significant correlation (*P* < 0.01), and one star indicates that indexes showed a significant correlation (*P* < 0.05). The brightness of the red or blue color represents the value of the correlation coefficient.

The results of the correlation analysis for the process variables and morphological parameters are set out in Figure 4. CAT, SOD, and SP had significant positive correlation with all morphological parameters (SL, RL, RGW, GD, SGW). The correlation coefficient of CAT and morphological parameters were greater than 0.80. However, PRO and MDA were significantly negative correlated with all morphological parameters, and the correlation coefficient were more than −0.75 except for MDA with GD (−0.40). SS had significant negative correlation with only SL and SGW, while CHL was not correlated with all virtually morphological parameters.

### SE of Different Tree Species on *A. pedunculata*

**FIGURE 5:**
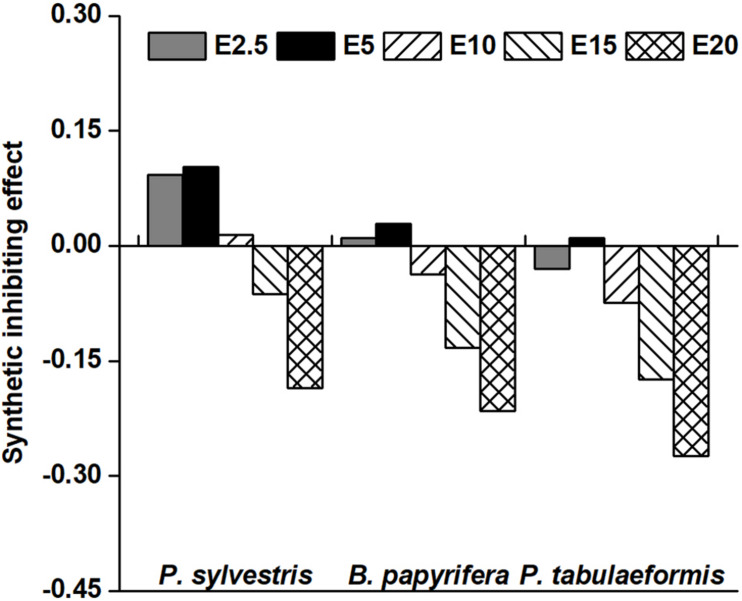
Synthetic inhibiting effect of the aqueous leaf extracts from the donor species on the target *Amygdalus pedunculata* of YC-1. The synthetic inhibiting effect (SE) value was calculated as the average allelopathy index (*RI*) value of all indicators of each treatment of the target *A. pedunculata* species of YC-1. The concentrations of 0% (CK), 2.5% (E2.5), 5% (E5), 10% (E10), 15% (E15), and 20% (E20) of aqueous leaf extracts from the donor species—*Pinus sylvestris* (*P. sylvestris*), *Broussonetia papyrifera* (*B. papyrifera*), and *Pinus tabulaeformis* (*P. tabulaeformis*).

Twenty-two indicators, including GP, GR, GI, SL, RL, SFW, RFW, SDW, RDW, GD, enzyme activity and so on, were used to analyze the allelopathy SE value to reflect the total allelopathic intensity of four species of tree aqueous leaf extracts on YC-1. Aqueous leaf extracts of *P. sylvestris* and *B. papyrifera* at concentrations of E2.5 and E5 were positive and had promotional effects on YC-1 (Figure 5). At the concentration of E5, the SE values of YC-1 peaked at 0.10 and 0.03. In addition, SE values of *P. tabulaeformis* aqueous leaf extracts at all tested concentrations on YC-1 was negative (Except E10), indicating an inhibitory effect. The SE values of the three species of tree aqueous leaf extracts on YC-1 were as follows: *P. sylvestris* > *B. papyrifera* > *P. tabulaeformis*.

## Discussion

Seed germination is a key stage related to the natural renewal, reproduction and survival of plant populations. The germination potential can reflect the uniformity of germination and the strength of vitality, the germination rate can reflect the quantity and quality of seed germination, and the germination index reflects the deterioration of seeds, which can reflect the germination ability and vitality of seeds (Siri-Udom et al., 2017). We observed a significant reduction in the seed germination and seedling growth of *A. pedunculata* as concentrations increased in our study. This result was supported by previous studies that have found that a concentration gradient of active substances had an important effect on plant growth (Einhellig, 1986; Timmerman, 2003; An et al., 2015; Sitthinoi et al., 2017). This study indicated that the aqueous leaf extracts of *Pinus sylvestris*, *Broussonetia papyrifera*, and *Pinus tabulaeformis* could promote *A. pedunculata* seed germination and seedling growth compared with *P. simonii* treatment under lower concentrations (E2.5 and E5). *P. simonii* aqueous leaf extracts induced the lowest seed germination and seedling growth of YC-1 and SC-6, and mortality was common in YC-1 at highest concentrations (E20). *P. simonii* was the worst for *A. pedunculata* seed germination. Therefore, we removed *P. simonii* from our follow-up experiment. The YC-1 seed germination process represents a more sensitive state than SC-6 when different donor leaf extract was added; at the same time, the effect of YC-1 seed germination was more obvious. Luo et al. (2017) proposed that low concentrations (25 and 50 g mL^–1^) of foliage litter of *Artemisia halodendron* had positive effects on the GR of a soil seed bank which is consistent with our findings. In addition, *Artemisia halodendron* litter had a minimal induction effect on seedling growth at high concentrations (100 and 200 g mL^–1^), indicating that the high concentration of *A. halodendron* litter strongly inhibited seed germination and seedling establishment (Luo et al., 2017).

The composition of tree species and the concentration of stimulant containing different quantities of allelochemicals affects the process of seed germination and seedling growth and thus the growth condition of *A. pedunculata* (Deng et al., 2017). The allelopathic effect is strongly related to the allelochemicals of donor and plant-environment interactions (Blanco, 2007). Most of the allelochemicals of the donor are secondary metabolites, which affect the growth and development of the recipient plants through leaching, volatilization, root exudates, plant residues, and decomposition of surface litters (Koocheki et al., 2013). Because the allelochemicals in the donor plants often consist of a variety of allelochemicals, their allelochemicals can show synergistic, additive, or even antagonistic effects on the same recipient plant (Zhang et al., 2016; Alexa et al., 2018). This finding is consistent with those of Kayanifard and Mohsenzadeh (2017), who suggested that the extracts of four ecotypes of *Ajowan* seeds differed in the strength of their allelopathic effects on seed germination, seedling growth, proline, and sugar content, which may be related to the identity of the allelochemicals (thymol, c-terpinene, and p-cymene) contained in the seeds.

Differences in the seedling growth of *A. pedunculata* observed in the laboratory and pot experiments might be attributed to several environmental factors, such as temperature, humidity, light intensity, quantity of nitrogen dioxide, nutrient levels, and breathability. Additional trials exploring the effects and underlying mechanisms of the extracts of the three tree species on the seedling growth of YC-1 are needed to identify a mixed configuration mode that would be superior for the growth of *A. pedunculata*.

As the concentration of tree leaf extracts increased, the CAT content of *A. thrpedunculata* seedlings first increased and then decreased, while POD and SOD activity decreased gradually, indicating that the ability of the protective enzyme system to remove active oxygen gradually decreased. Our results are consistent with those of Roshchina and Roshchina (1993), who showed that when plants encounter high concentrations of allelochemicals, O_2_^–^ production accelerates. As a consequence, the balance in active oxygen metabolism is destroyed, MDA content and membrane permeability in plants increase, and activities of protective enzymes (e.g., SOD, POD, and CAT) are inhibited, thus leading to a reduction in plant growth indicators. Many studies have also shown that some phenolic substances in allelopathic substances can inhibit enzyme activity, reduce the scavenging effect of reactive oxygen species, and damage the structure of the plant cell membrane, thereby weakening the protection provided to plant tissues (Zeng et al., 2001; Al Harun et al., 2014).

Malondialdehyde, SS, CMP, and PRO increased—and SP decreased—as the concentrations of tree leaf extract increased, indicating that a decrease in enzyme activity led to increases in membrane permeability and an enhancement of the stress tolerance of *A. pedunculata* seedlings. Changes in SP will affect the permeability of the cell membrane, the transport of substances, and the conversion of energy related to membrane function (Sunmonu and Van Staden, 2014; Bouhaouel et al., 2018). Under E2.5 and E5, SS in the seedlings of YC-1 was lower, indicating that the degree of stress was lower. However, SS in the seedlings of YC-1 was higher at higher concentrations (E15 and E20), indicating that the seedlings of *A. pedunculata* could improve their stress resistance by accumulating more sugar. SP in the seedlings of YC-1 decreased gradually as the concentrations of the aqueous tree leaf extracts of the three tree species increased, especially at high concentrations (E15 and E20). In addition, as the SP of YC-1 seedlings decreased, protein production became increasingly inhibited. Our findings are consistent with those of Bradford (1976), who proposed that under adverse conditions, plants would actively accumulate SS, thereby increasing their resistance to stress. SP is closely tied to enzyme activity and functions in various metabolic processes in plants, including photosynthesis.

As the concentrations of leaf extract increased, SP, CHL, and RA decreased, indicating that high concentrations of these compounds in the extract led to a greater degree of inhibition of the growth of *A. pedunculata*. Allelochemicals can directly affect photosynthesis by regulating physiological and metabolic activities or indirectly by altering chlorophyll synthesis (Pedrol et al., 2000). RA refers to the strength of plant root metabolism and nutrient and water absorption capacity. Therefore, the amount of root activity has a critical impact on plant growth and development (Maxwell and Johnson, 2000). There were no significant differences in the chlorophyll content between leaf extracts of *B. papyrifera* and *P. tabulaeformis* on YC-1 seedlings for concentrations of E2.5, E5, and E10. In addition, the aqueous leaf extract of the three tree species had a significant promoting effect on the RA of seedlings, indicating that at low concentrations, *A. pedunculata* seedlings could carry out normal levels of photosynthesis and complete all of their primary physiological and metabolic functions. The effects of aqueous leaf extracts of *B. papyrifera* and *P. tabulaeformis* on the chlorophyll content and RA of YC-1 seedlings were lower than that of *P. sylvestris* at high leaf extract concentrations.

The four tree species tested in this study produced seed germination and seedling growth stimulants, although the production of these stimulants varied among laboratory and pot experiments. Under low population densities of *A. pedunculata* in arid and semi-arid region with low annual rainfall in northwest China, *P. sylvestris and B. papyrifera* could be used to promote the growth of *A. pedunculata* seedlings. Such trees can facilitate the settlement of *A. pedunculata* seedlings and increase the survival of *A. pedunculata* seedlings. Generally, our study has laid a foundation from which a planting allocation model of artificial mixed forest in arid and semi-arid region could be developed. Furthermore, our study provides a new perspective on how stereo-complex ecosystems could be created. Vegetation reconstruction is conducive to the restoration of eco-economic forests of *A. pedunculata*, which can not only provide ecological benefits but also increase the income of local residents.

We plan to establish a three-dimensional composite configuration model of trees and shrubs (*P. sylvestris* + *A. pedunculata* and *B. papyrifera* + *A. pedunculata*) at a local field test site to evaluate the effect of local natural conditions on plant growth, soil biological communities, physical and chemical properties, and other indicators as well as assess the possibility of field cultivation.

## Conclusion

This study showed that different *A. pedunculata* varieties (YC-1 and SC-6) had various sensitivities to the allelochemicals of donor species (*P. sylvestris*, *B. papyrifera*, *P. simonii*, and *P. tabulaeformis*), which could stem the type, quantity, concentrations, and the characteristics of allelopathic substances produced by these trees. The obtain results proved that the seed germination and seedling growth of YC-1 and SC-6 were higher when plants were treated with the aqueous leaf extracts of *P. sylvestris*, *B. papyrifera*, and *P. tabulaeformis* than the aqueous leaf extracts of *Populus simonii* at concentrations of 2.5% (E2.5) and 5% (E5). Furthermore, the donor leaf extract was more sensitive to YC-1 than to SC-6, Therefore, *P. simonii* and SC-6 were excluded in subsequent analyses. Considering their allelopathic effects, *P. sylvestris* and *B. papyrifera* should be planted with *A. pedunculata* at low population densities. The results provide new insight into creating stereo-complex ecosystems (*P. sylvestris* + *A. pedunculata* and *B. papyrifera* + *A. pedunculata*) in arid and semi-arid region with low annual rainfall in northwest China. Additional study under field conditions is necessary for evaluating the effect of local natural conditions on plant growth, soil biological communities, physical and chemical properties, and other indicators as well as discuss the situation of field cultivation.

## Data Availability Statement

The original contributions presented in the study are included in the article/Supplementary Material, further inquiries can be directed to the corresponding author.

## Author Contributions

XW and JW designed the research. RZ and AS detected samples in the lab. XW participated in the experimental design. XW wrote the manuscript. LD and HW provided experimental materials and field investigations. JW, XW, and RZ revised the manuscript. All authors approved the final manuscript.

## Conflict of Interest

The authors declare that the research was conducted in the absence of any commercial or financial relationships that could be construed as a potential conflict of interest.

## Abbreviations

CAT: activities of catalase
CHL: chlorophyll
CK: 0%
CMP: cell membrane permeability
E2.5: 2.5%
E5: 5%
E10: 10%
E15: 15%
E20: 20%
GD: ground diameter
GI: germination index
GP: germination potential
GR: germination rate
MDA: malondialdehyde
POD: peroxidase
PRO: proline
RA: root activity
RDW: root dry weight
RFW: root fresh weight
RL: root length
SC: Shenmu County
SDW: seedling dry weight
SFW: seedling fresh weight
SL: seedling length
SOD: superoxide dismutase
SP: soluble protein
SS: soluble sugar
YC: Yuyang County.
