# Supplementary material for: The Effects of Leaf Extracts of Four Tree Species on Amygdalus pedunculata Seedlings Growth
Source: Front Plant Sci. 2021 Jan 13;11:587579. doi: 10.3389/fpls.2020.587579 (PMC7873849; doi:10.3389/fpls.2020.587579)
Supplement: Supplementary file 1 [file Data_Sheet_1.docx]

Supplementary Tables

**Supplementary Table 1.** Effect of different concentrations of the aqueous leaf extracts of four tree species on the seed germination of *Amygdalus pedunculata* of YC-1. (Mean ±Standard Deviation, n=4)

| **Tree species** | **Concentrations**  **(%)** | **Indicators (YC-1)** | | |
| --- | --- | --- | --- | --- |
|  |  | **Germination potential** | **Germination rate** | **Germination index** |
|  |  | **(%)** | **(%)** |  |
| *Pinus* | CK | 61.00±3.82b | 81.00±3.82b | 38.42±0.74c |
| *sylvestris* | E2.5 | 68.00±3.26a | 93.00±2.00a | 49.26±1.89a |
|  | E5 | 60.00±3.26b | 88.00±3.26a | 43.91±1.95b |
|  | E10 | 50.00±5.16c | 77.00±3.82b | 37.69±1.95c |
|  | E15 | 46.00±5.16c | 66.00±5.16c | 31.28±2.26d |
|  | E20 | 36.00±3.26d | 56.00±3.26d | 20.71±1.42e |
| *Broussonetia* | CK | 61.00±3.82b | 81.00±3.82a | 38.42±0.74a |
| *papyrifera* | E2.5 | 72.00±3.26a | 81.00±6.83a | 37.87±1.83a |
|  | E5 | 64.00±3.26b | 77.00±3.82a | 34.48±3.13b |
|  | E10 | 55.00±3.82c | 75.00±3.82a | 32.00±1.50b |
|  | E15 | 33.00±3.82d | 66.00±5.16b | 25.46±1.82c |
|  | E20 | 28.00±3.26d | 61.00±2.00b | 21.7±1.97d |
| *Populus* | CK | 61.00±3.82b | 81.00±3.82a | 38.42±0.74b |
| *simonii* | E2.5 | 66.00±2.3a | 76.00±5.65a | 41.19±1.71a |
|  | E5 | 56.00±3.26c | 65.00±3.82b | 34.72±0.83c |
|  | E10 | 44.00±3.26d | 58.00±2.30c | 28.80±1.79d |
|  | E15 | 39.00±3.82e | 52.00±3.26d | 22.99±1.20e |
|  | E20 | N | N | N |
| *Pinus* | CK | 61.00±3.82b | 81.00±3.82c | 38.42±0.74c |
| *tabulaeformis* | E2.5 | 80.00±3.26a | 92.00±3.26a | 46.74±0.86a |
|  | E5 | 80.00±5.65a | 87.00±2.00b | 46.87±1.07a |
|  | E10 | 59.00±2.00b | 76.00±3.26d | 41.86±2.02b |
|  | E15 | 42.00±5.16c | 71.00±3.82e | 29.89±2.85d |
|  | E20 | 34.00±2.30d | 52.00±3.26f | 21.76±2.21e |

CK, 0%; E2.5, 2.5%; E5, 5%; E10, 10%; E15, 15%; E20, 20%. N means no value (the seeds did not germinate and died). Different lowercase letters among treatments meant significant difference at 0.05 level.

**Supplementary** **Table 2.** Effect of different concentrations of the aqueous leaf extracts of four tree species on the seed germination of *Amygdalus pedunculata* of SC-6. (Mean ±Standard Deviation, n=4)

| **Tree species** | **Concentrations**  **(%)** | **Indicators (SC-6)** | | |
| --- | --- | --- | --- | --- |
|  |  | **Germination potential** | **Germination rate** | **Germination index** |
|  |  | **(%)** | **(%)** |  |
| *Pinus* | CK | 66.00±5.16a | 83.00±3.82a | 38.68±2.31b |
| *sylvestris* | E2.5 | 62.00±5.16ab | 75.00±5.03b | 43.83±3.77a |
|  | E5 | 57.00±3.82b | 62.00±2.30c | 34.10±1.32c |
|  | E10 | 50.00±5.16c | 56.00±3.26d | 30.88±1.26c |
|  | E15 | 46.00±5.16c | 46.00±5.16e | 26.55±2.96d |
|  | E20 | 36.00±3.26d | 36.00±3.26f | 19.23±1.38e |
| *Broussonetia* | CK | 66.00±5.16a | 83.00±3.82a | 38.68±2.31a |
| *papyrifera* | E2.5 | 64.00±3.26a | 83.00±3.82a | 37.84±2.63a |
|  | E5 | 51.00±3.82b | 61.00±3.82b | 26.33±1.73b |
|  | E10 | 48.00±3.26bc | 73.00±3.82c | 31.39±0.51c |
|  | E15 | 43.00±3.82c | 50.00±2.30d | 20.24±3.41d |
|  | E20 | 30.00±2.3d | 37.00±5.03e | 14.44±2.39e |
| *Populus* | CK | 66.00±5.16a | 83.00±3.82a | 38.68±2.31a |
| *simonii* | E2.5 | 65.00±3.82a | 76.00±5.65b | 39.3±2.39a |
|  | E5 | 54.00±2.30b | 66.00±2.30c | 28.35±2.99b |
|  | E10 | 41.00±2.00c | 59.00±2.01d | 26.52±1.38b |
|  | E15 | 33.00±3.82d | 53.00±2.00e | 19.07±2.25c |
|  | E20 | 28.00±3.26d | 47.00±2.00f | 17.80±2.10c |
| *Pinus* | CK | 66.00±5.16b | 83.00±3.82b | 38.68±2.31b |
| *tabulaeformis* | E2.5 | 70.00±5.16b | 85.00±6.00ab | 39.34±2.00b |
|  | E5 | 80.00±3.26a | 93.00±6.83a | 49.52±1.64a |
|  | E10 | 64.00±3.26b | 81.00±3.82b | 40.55±1.58b |
|  | E15 | 41.00±2.00c | 71.00±6.00c | 28.56±3.95c |
|  | E20 | 37.00±3.82c | 55.00±7.57d | 20.89±3.28d |

CK, 0%; E2.5, 2.5%; E5, 5%; E10, 10%; E15, 15%; E20, 20%. Different lowercase letters among treatments indicate significant difference at the 0.05 level.

**Supplementary Table 3.** Effect of different concentrations of the aqueous leaf extracts of three trees species on physiological and biochemical indicators of *Amygdalus pedunculata* of YC-1. (Mean ±Standard Deviation, n=4)

| **Trees species** | **Concentrations**  **(%)** | **Indicators (YC-1)** | | | | | | |
| --- | --- | --- | --- | --- | --- | --- | --- | --- |
|  |  | **Content of malondialdehyde** | **Content of soluble sugar** | **Cell membrane permeability** | **Content of proline** | **Content of soluble protein** | **Content of chlorophyll** | **Root activity** |
|  |  | **(mmol·g^-1^FW)** | **(mg·g^-1^FW)** | **(%)** | **(mg·g^-1^FW)** | **(mg·g^-1^FW)** | **(mg·g^-1^FW)** | **(mg·g^-1^·min^-1^)** |
| *Pinus* | CK | 4.98±1.92c | 26.14±0.88f | 64.75±1.09f | 0.02±0.01e | 28.02±5.98a | 4.33±0.01a | 3.74±2.16ab |
| *sylvestris* | E2.5 | 4.60±0.86c | 31.83±1.10e | 79.25±4.09e | 0.02±0.02e | 26.75±0.96ab | 2.28±0.11b | 3.95±0.40ab |
|  | E5 | 5.55±0.73c | 37.53±1.07d | 90.96±3.13d | 0.02±0.01d | 23.60±0.66bc | 2.09±0.05c | 4.54±0.36a |
|  | E10 | 6.16±0.36bc | 40.74±0.62c | 96.96±1.52c | 0.03±0.01c | 21.94±2.49c | 1.84±0.05d | 3.40±0.21ab |
|  | E15 | 7.64±0.90ab | 45.39±1.72b | 101.01±2.52b | 0.03±0.01b | 16.94±0.67d | 1.67±0.01e | 2.84±0.04b |
|  | E20 | 8.63±1.62a | 49.2±0.23a | 106.16±0.32a | 0.04±0.01a | 11.99±1.01e | 1.62±0.04e | 2.54±0.46b |
| *Broussonetia* | CK | 4.98±1.92b | 26.14±0.88f | 64.75±1.09f | 0.02±0.01c | 28.02±5.98a | 4.33±0.01a | 3.74±2.16ab |
| *papyrifera* | E2.5 | 4.83±1.17b | 31.72±0.44e | 81.25±0.28e | 0.02±0.01e | 25.91±1.22ab | 2.07±0.02b | 3.85±0.11ab |
|  | E5 | 5.22±0.99b | 34.09±1.73d | 93.07±0.60d | 0.02±0.01c | 19.47±8.55bc | 1.85±0.02c | 4.36±0.33a |
|  | E10 | 6.17±0.89b | 39.82±1.70c | 97.62±2.20c | 0.03±0.03b | 16.99±0.60b | 1.75±0.18c | 3.37±0.25ab |
|  | E15 | 6.66±0.81ab | 44.87±0.51b | 101.22±0.32b | 0.05±0.02a | 13.32±0.78bc | 1.63±0.04d | 2.82±0.14b |
|  | E20 | 8.09±0.87a | 48.4±0.68a | 106.8±2.79a | 0.05±0.01a | 9.18±0.97c | 1.52±0.07d | 2.49±0.09b |
| *Pinus* | CK | 4.98±1.92e | 26.14±0.88f | 64.75±1.09e | 0.02±0.02f | 28.02±5.98a | 4.33±0.01a | 3.74±2.16ab |
| *tabulaeformis* | E2.5 | 5.18±0.95e | 27.31±0.70e | 83.77±4.63d | 0.02±0.01e | 23.63±0.21b | 2.00±0.04b | 3.80±0.19ab |
|  | E5 | 7.34±0.42d | 31.27±0.85d | 93.59±3.27c | 0.02±0.01d | 17.66±0.20c | 1.82±0.02c | 4.30±0.82a |
|  | E10 | 9.64±0.56c | 38.42±0.71c | 98.82±1.15b | 0.03±0.02c | 13.75±1.55d | 1.71±0.09d | 3.30±0.03ab |
|  | E15 | 15.37±0.86b | 43.27±0.83b | 101.38±2.97b | 0.03±0.01b | 9.81±0.51e | 1.62±0.04e | 2.73±0.05bc |
|  | E20 | 23.37±1.26a | 47.64±0.36a | 108.69±4.69a | 0.05±0.01a | 5.89±0.60f | 1.51±0.04f | 2.20±0.07c |

CK, 0%; E2.5, 2.5%; E5, 5%; E10, 10%; E15, 15%; E20, 20%. Different lowercase letters among treatments indicate significant difference at the 0.05 level.
